# Malaria reemergence in the Peruvian Amazon region.

**DOI:** 10.3201/eid0502.990204

**Published:** 1999

**Authors:** J Aramburú Guarda, C Ramal Asayag, R Witzig

**Affiliations:** Loreto Department of Public Health, Iquitos, Peru. jaramburu@oge.sld.pe

## Abstract

Epidemic malaria has rapidly emerged in Loreto Department, in the Peruvian Amazon region. Peru reports the second highest number of malaria cases in South America (after Brazil), most from Loreto. From 1992 to 1997, malaria increased 50-fold in Loreto but only fourfold in Peru. Plasmodium falciparum infection, which has increased at a faster rate than P. vivax infection in the last 3 years, became the dominant Plasmodium infection in the highest transmission areas in the 1997 rainy season. The vector Anopheles darlingi has also increased during this epidemic in Loreto. Moreover, chloroquine and pyrimethamine-sulfadoxine drug-resistant P. falciparum strains have emerged, which require development of efficacious focal drug treatment schemes.


### Malaria Reemergence in the Peruvian Amazon Region

**Javier Aramburú Guarda,* César Ramal Asayag,* and Richard Witzig†**
*Loreto Department of Public Health, Iquitos, Peru; and †Centers for Disease Control and Prevention, Atlanta, Georgia,USA

Epidemic malaria has rapidly emerged in Loreto Department, in the Peruvian Amazon region. Peru reports the second highest number of malaria cases in South America (after Brazil), most from Loreto. From 1992 to 1997, malaria increased 50-fold in Loreto but only fourfold in Peru. *Plasmodium falciparum* infection, which has increased at a faster rate than *P. vivax* infection in the last 3 years, became the dominant *Plasmodium* infection in the highest transmission areas in the 1997 rainy season. The vector *Anopheles darlingi* has also increased during this epidemic in Loreto. Moreover, chloroquine and pyrimethamine-sulfadoxine drug-resistant *P. falciparum* strains have emerged, which require development of efficacious focal drug treatment schemes.

The Peruvian Department of Loreto is the last part of the greater Amazon region to report the reemergence of epidemic malaria after the eradication efforts of the 1960s. Loreto, which comprises nearly one-fourth of the land mass of Peru, has the ecologic characteristics of the Amazon lowlands (Figure 1). In central Loreto, the Marañon and Ucayali Rivers combine to form the nascent Amazon River. Iquitos (population 345,000), the only large urban center in Loreto, is accessible only by air or river. The rural population of 474,000 is clustered in towns and villages throughout the Amazon tributary system. Loreto's economy relies on basic agriculture, fishing, lumber, commercial activities, and petroleum.

### Epidemiology

Since 1941, the highest number of malaria cases in Peru was 95,000 in 1944, and the lowest was 1,500 cases in 1965 (after the malaria eradication campaign) (1). *Plasmodium falciparum* infections (chloroquine-sensitive) were confined to the northwestern coastal areas of Peru bordering Ecuador, with occasional reports of cases from the north and eastern Loreto border areas with Ecuador, Colombia, and Brazil.

Address for correspondence: Javier Aramburú Guarda, Oficina General de Epidemiología, Ministerio de Salud, Perú, Avenida Camilo Carrillo 402, Jesús María, Lima11, Perú; e-mail: jaramburu@oge.sld.pe.

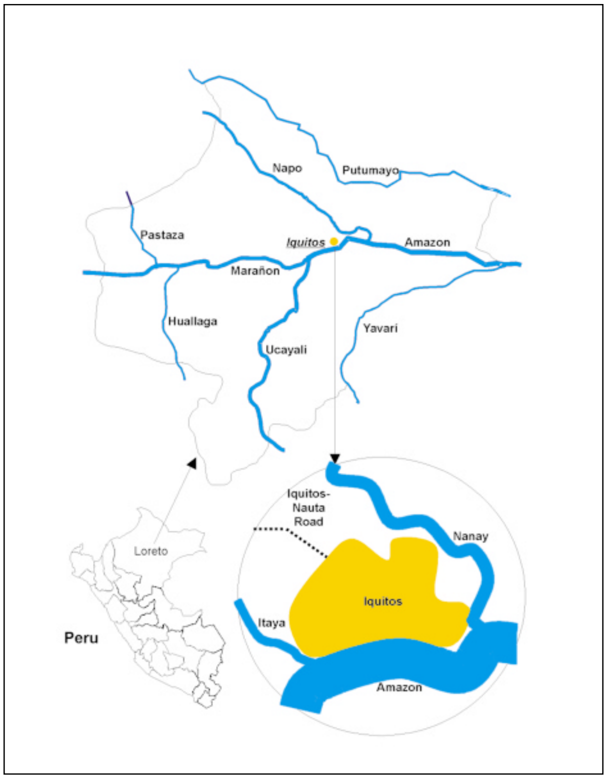

Figure 1: The department of Loreto and the city of Iquitos in Peru.

In 1988, no cases of *P. falciparum* were reported in Loreto. In 1991, 140 cases were reported; the number increased annually until 1997 when 54,290 slide-confirmed *P. falciparum*

cases and 85 deaths were reported (Figure 2). In 1997, a total of 121,268 malaria cases were reported in Loreto; the number indicates an equally dramatic rise in *P. vivax* malaria. *P. malariae* is infrequently reported (44 cases in 1997). An additional 36,864 persons were treated for probable malaria (slide-negative or no available laboratory diagnosis) in 1997. Therefore, the number of malaria cases in 1997 reported by slide or clinical definition was 158,115 (2). More *Plasmodium* infections occurred in males (60.5%) than in females (39.5%). Malaria transmission has been seasonal in the Loreto epidemic, with peaks in the rainy season from November to June (Figure 3).

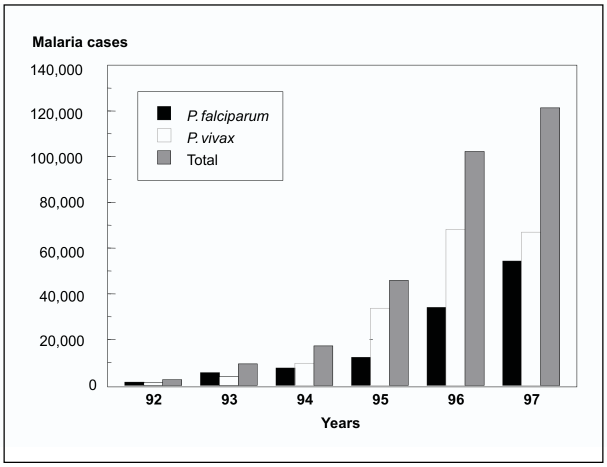

Figure 2: Malaria incidence in Loreto, 1992-1997.

Malaria around the city of Iquitos accounts for most cases in Loreto. Indigenous *P. vivax* malaria was initially reported in the Iquitos area (Rumococha and Zungarococha) in April 1991, and *P. falciparum* was first detected (Padrecocha) in November 1994. A small number of *P. falciparum* cases have been diagnosed in patients who had not traveled outside Iquitos in the 2 months before illness. Entomologic investigations have confirmed that malaria transmission inside Iquitos is possible. Two high transmission zones surround Iquitos: communities on the Nanay River (which empties into the Amazon after bordering northern and eastern Iquitos) and communities along the first asphalt extension road from Iquitos (the unfinished Iquitos-Nauta road). Two additional high transmission zones include communities on the Yavarí River and the Pastaza River. The annual parasite index (number of malaria cases per 1,000 persons per year), with malaria transmission by districts in Loreto, is shown in Figure 4. The global annual parasite index for Loreto for 1997 was 148 per 1,000 persons.

Age-specific attack rates of *P. falciparum* in most of Loreto are consistent with hypoendemic malaria (defined as parasite or spleen rates less than 10% [Figure 5A]), although in 1997 in the highest transmission areas, mesoendemic malaria measurements were transiently documented in prevalence studies (11% to 50% parasite rates) (3). The high parasitemia rates in adults in Loreto emphasize the environmental

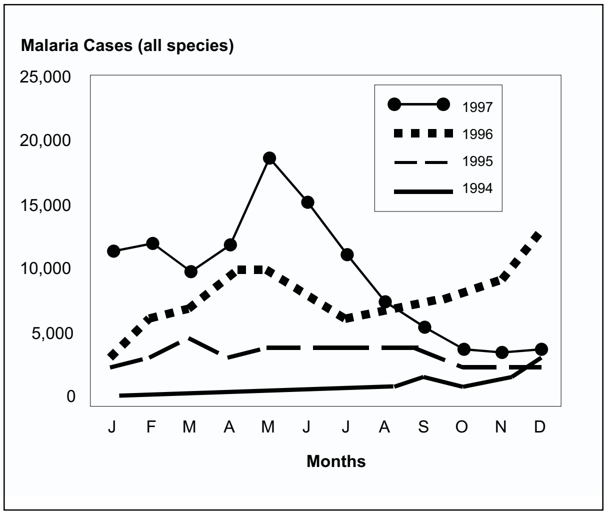

Figure 3. Malaria (all species) incidence in Loreto, by month, 1994–1997.

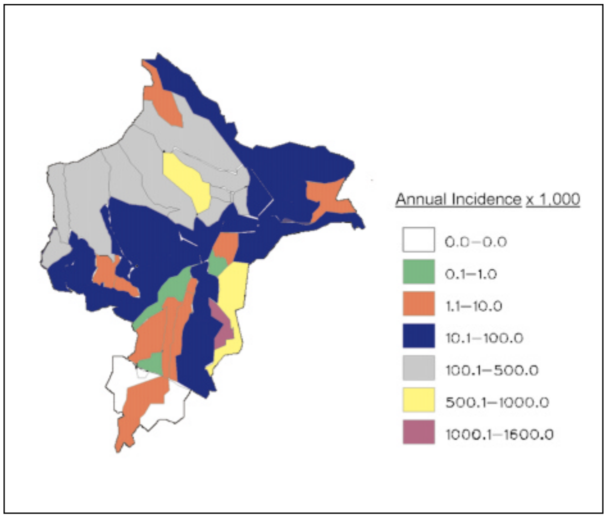

Figure 4: Annual parasite index in Loreto, by district (per 1,000 population).

risks for malaria infection to adults and the lack of immunity in this epidemic. Age-specific death rates have documented a substantial risk for death among those under 5 years of age and among those 60 years of age and older, although the number of deaths in children under 5 years declined in 1997 (Figure 5B). The death rate from *P. falciparum* malaria has been decreasing (from 1.8 to 1.3 per 1000) in the last 4 years (1994 to 1997).

Because of the current epidemic, Peru reports the second highest number of malaria cases in South America (after Brazil)—most from Loreto (in 1997, Loreto reported 67.2% of the malaria cases in Peru) (4). From 1992 to 1997, malaria increased 50-fold in Loreto and fourfold in Peru. In 1992, 1.6% of malaria cases in Peru were due to *P. falciparum*; in 1997 (in Loreto), 44.8% of malaria cases were due to *P. falciparum* (5,6).

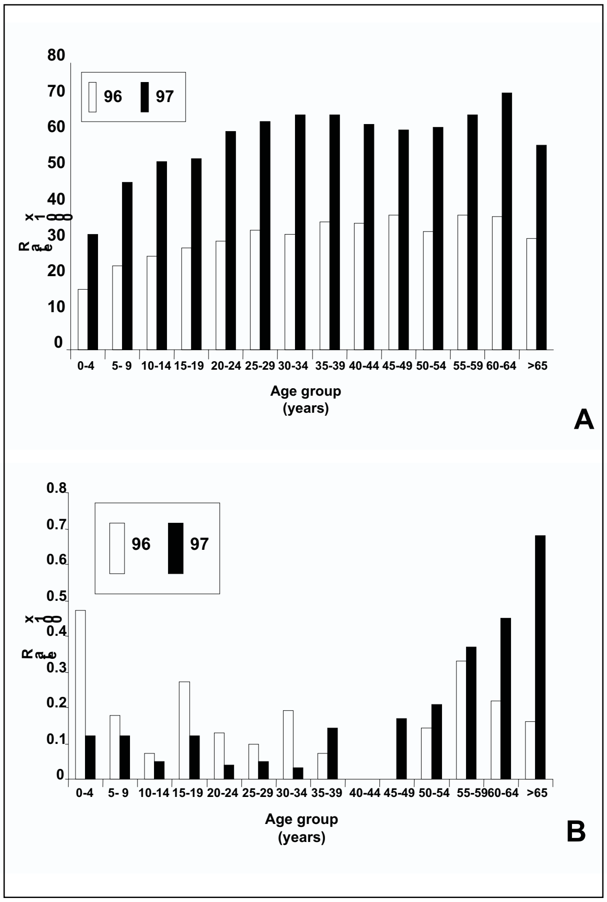

Figure 5. A. Age-adjusted incidence rates for *Plasmodium falciparum* malaria in Loreto, 1996-1997. B. Age-adjusted death rates for *Plasmodium falciparum* malaria in Loreto, 1996-1997.

### Vector Dynamics

Paralleling the malaria epidemic has been an increase in the highly competent and anthropophilic malaria vector, *Anopheles darlingi*, the principal *P. falciparum* vector in the Brazilian Amazon (7,8). *An. darlingi* was not found in the Iquitos area in 1991 (9) when indigenous *P. vivax* was detected but has now been found in all areas of Loreto and even within Iquitos (10-12). The abundance of *An. darlingi,* already established when *P. falciparum* was first reported from the peri-Iquitos area in 1994, is clearly linked to the marked rise in *P. falciparum* transmission.

*An. darlingi* larvae habitats around Iquitos include small pools on cleared land, fish hatcheries, areas of poor sanitation, swamps, and the edges of small rivers. *An. darlingi* in the Iquitos area bite indoors or outdoors from dusk to midnight, with a small dawn peak. *An. darlingi* represent more than 90% of the anophelines in the peri-Iquitos area in the rainy season; they decrease but remain the major anopheline species in the dry season.

*An. benarrochi* is the dominant malaria vector in western Loreto, while *An. triannulatus* is the dominant vector in eastern Loreto. Other *Anopheles* species in Loreto known to be malaria vectors are *An. oswaldoi, An. nuneztovari,* and *An. rangeli.*

### Climatic Associations

Loreto has a climate typical of the Amazon region, with a rainy season from November to May and a second precipitation peak in September in the dry season. The El Niño phenomenon in 1997 extended the dry season in Loreto, but caused torrential rains along coastal Peru.

The level of the Amazon River in Iquitos varies 10 meters annually (from 108 to 118 meters above sea level); flooding is usual on the smaller tributaries but infrequent on the main Amazon River. The mean annual temperature is 28ºC, the warmest months are September and October, and the humidity is persistently higher than 87% (2).

Climatic indexes of river level, rainfall, temperature, and humidity at Iquitos were analyzed to determine association with malaria prevalence. The height of the Amazon River at Iquitos depends on rainfall on the eastern side of the Andes Mountains as well as precipitation in Loreto. The height of the Amazon is related to malaria prevalence; the highest river level precedes the malaria prevalence peak by 2 months, and the lowest level precedes the malaria prevalence low by 2 months (Figure 6A). Precipitation peaked twice in 1997: 3 months before and the same month that malaria cases reached their highest number (Figure 6B). The mean temperature was 27°C to 29˚C and was inversely associated with malaria cases (Figure 6C). Relative humidity was 87% to 93% and was not associated with malaria prevalence.

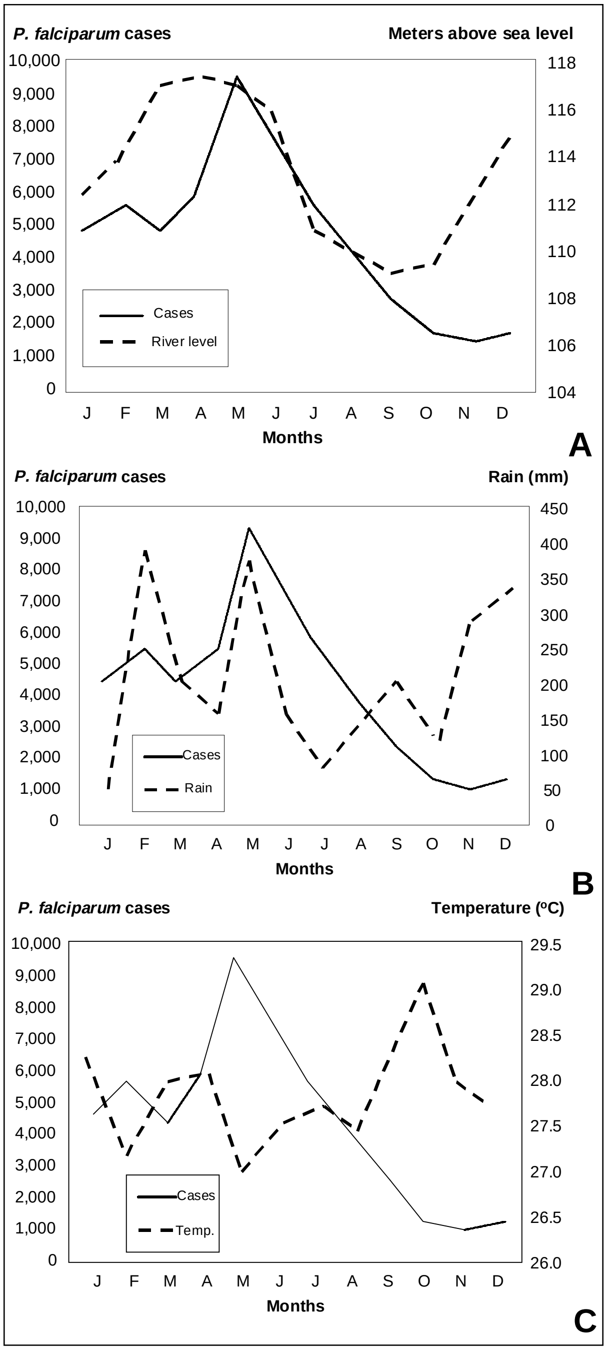

Figure 6: *Plasmodium falciparum* malaria incidence in Loreto. A. Average Amazon River level at Iquitos, by month. B. Precipitation at Iquitos, by month. C. Average temperature (°C) at Iquitos, by month.

### Drug Resistance and Drug Treatment

Clinical resistance of *P. falciparum* parasites to chloroquine and to the combination antifolate drug pyrimethamine-sulfadoxine is a growing public health problem in Peru. In an epidemic characterized by limited population immunity, parasitologic resistance (persistence or reemergence of parasites) inevitably leads to clinical resistance (reemergence of malarial symptoms).

Multiple regional in vivo drug susceptibility testing for chloroquine (10% to 70% resistance) and pyrimethamine-sulfadoxine (10% to 63% resistance) has documented significant focal differences in resistance patterns (13-15). Three *P. falciparum* strains have converged on Loreto and Iquitos: (Brazilian-pyrimethamine-sulfadoxine and chloroquine, complete resistance; Loretana–pyrimethamine-sulfadoxine, variable resistance; chloroquine resistant; and Pastazan/coastal-chloroquine susceptible). These strains were likely introduced and disseminated in different ways (e.g., routine travel, illegal narcotrafficking), but the increased abundance of *An. darlingi* in Loreto has allowed them to thrive. The highest percentage of strains resistant to multiple drugs (both chloroquine and pyrimethamine-sulfadoxine–resistant) have been found in the border areas of Colonia Angamos on the Yavarí River, communities on the Blanco River, and in Santa Clara near Iquitos.

From July through November 1996, we performed in Iquitos two prospective studies of *P. falciparum* drug resistance in patients from periurban and rural areas in Loreto. Twenty-eight day in vivo drug susceptibility testing of *P. falciparum* to pyrimethamine-sulfadoxine was performed in 62 patients. The WHO in vitro microtest method was used to evaluate *P. falciparum* susceptibility to mefloquine, chloroquine, pyrimethamine-sulfadoxine, and quinine in pretreatment specimens from the same patients (16). Results showed significant pyrimethamine-sulfadoxine resistance in Loreto (31% in vivo and 67% in vitro) and documented in vitro resistance of chloroquine (78% resistance). *P. falciparum* parasites were susceptible to mefloquine and quinine (17).

A recent study in Loreto showed that in vivo resistance to pyrimethamine-sulfadoxine correlated with specific point mutations in *P. falciparum* dihydrofolate reductase at amino acid positions 51, 108, and 164, and dihydropteroate synthase mutations at positions 437, 540, and 581 (18). Further studies mapping the molecular resistance patterns of *P. falciparum* in Loreto are under way.

Malaria diagnosis and treatment are provided free of charge by the National Malaria Program. Focal treatment schemes have been designed depending on the local resistance patterns as documented by in vivo methods. Depending on local efficacy, first-line treatment of chloroquine, second-line of pyrimethamine-sulfadoxine, or third-line of quinine with clindamycin or tetracycline is administered (Figure 7). In 1998, treatment with mefloquine was initiated in selected communities with tertiary treatment schemes. All treatment lines include one dose of primaquine to eradicate *P. falciparum* gametocytes.

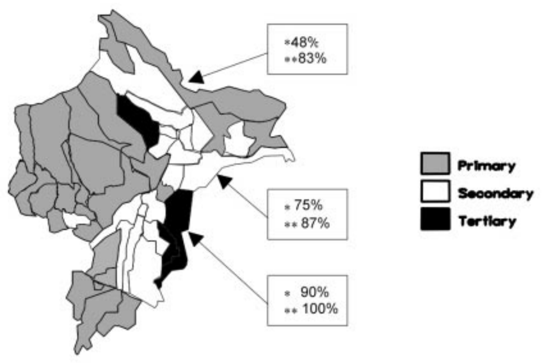

Figure 7: Initial *Plasmodium falciparum* malaria treatment schemes in Loreto by district, 1998. Treatment efficiency* (cases cured/cohort number x 100) and efficacy** (cases cured/[cases cured + resistant cases] x 100) shown for each treatment scheme region. Treatment schemes: Primary-chloroquine; secondary-pyrimethamine-sulfadoxine; tertiary-quinine.

### Control Schemes

Control strategies used by the Loreto Public Health Department and the National Malaria Program have included source, chemical, and biologic reduction strategies. Source reduction has consisted of community participation in identifying and filling in larval sites. Chemical control includes the use of insecticides in spatial fogging and domiciliary spraying and on bed nets. Pyrethroids (e.g., cyfluthrin) are used for fogging and domiciliary residual spraying. Since 1988, DDT has not been used in Loreto. An important challenge is that many Amazonian houses have open eaves and windows that allow mosquitoes to enter and exit without touching surfaces (except for a person's skin). *An. darlingi* behavioral studies have not yet been performed in Loreto to determine if the mosquitoes rest inside or outside after feeding. Larvaciding is performed with temephos (tetramethyl-thiodiphenylene phosphorothioate) placed at the surface of the larval breeding sites. Biologic control has been investigated with *Bacillus sphaericus* and *thuringiensis* in larval breeding pools. These bacteria have a short residual effect of 7 to 15 days in Loreto and require optimal conditions of shade for maximum efficacy.

Bed nets (*mosquiteros*) are ubiquitous in Loreto because of the many biting mosquito species. Local bed nets are made principally of cotton and sell for US$10 to $15. Insecticides were used in 1997 in the form of spraying (with cyfluthrin or deltamethrim) and impregnation (with either permethrim or deltamethrim) (19). Preliminary results show a decrease in malaria prevalence where insecticide-treated bed nets are in use.

### Conclusions

Malaria began to reemerge in the upper Pastaza River and eastern border areas of Loreto in the early 1990s. *P. falciparum* exceeded *P. vivax* infections in the early part of the Loreto epidemic (1992-1993). The abundance of *An. darlingi* starting in 1993 set the stage for explosive growth of both *P. falciparum* and *P. vivax* transmission, especially around Iquitos, which had previously not been affected. The increase in the number of malaria cases and the erosion of *P. vivax* dominance over *P. falciparum* occurred in spite of governmental interventions (treatment schemes, fumigation, larviciding, and domiciliary spraying). While *P. vivax* infections decreased slightly from 1996 to 1997, *P. falciparum* cases continued to increase by 60%. However, the rate of increase in total malaria cases slowed—from a factor of 2.23 from 1995 to 1996 to 1.19 in 1996 to 1997. The predominance of malaria infection in men is likely the result of occupational risks (e.g., working in recently cleared areas, logging, and night fishing and hunting).

The death rate of epidemic *P. falciparum* in Loreto appears to be low. The oft-quoted 1% death rate for *P. falciparum* infection (3) has been reduced seven times, perhaps because of determined efforts at the regional and national levels to provide rapid and appropriate treatment in malarious areas. Future efforts are planned to continue and expand coverage to further reduce deaths due to *P. falciparum*.

Although positive correlations were found between malaria transmission periods and Amazon River level and rainfall, the negative correlation with temperature may not be as significant as in other areas where higher temperatures are positively correlated with malaria cases (20). The mean temperature in the rainy season in Loreto is still within the optimal range for anopheline development, and so the observed temperature correlation may have little biologic influence on malarial prevalence.

Analysis of environmental factors indicates that the optimal timing of malaria control activities in 1998 was 2 months before the rainy season. Integrated malaria control activities began in the dry season with the strategy to intervene before high transmission season. In 1998, slide-documented malaria cases decreased 30.7% to 84,059, with *P. vivax* cases decreasing 15.3% to 56,710 and *P. falciparum* cases decreasing 49.6% to 27,336. The longer dry season in 1997, apparently caused by El Niño, may have assisted the Loreto Public Health Department and National Malaria Program efforts to control malaria in 1998.

Danger exists for further expansion of malaria in Loreto, especially through expansion of *An. darlingi* (into areas where it is not yet the dominant vector) and evolution of *P. falciparum* drug resistance. Consequently, more malaria control research is needed, particularly environmental control program studies, *Anopheles* behavioral studies, drug resistance testing, and community bed net trials.

### Acknowledgments

We thank Ernesto Colán Bernal, Jorge Quintana Zurita, and Mercy Panduro Gaviria for technical assistance for the in vitro drug resistance studies, Henrry Echeandía Rossell for computer design, Ernesto Berrocal Vilchez for entomologic coordination, Cristiam Carey Angeles and Carlos Calampa del Aguila for logistical support, and the workers at the Loreto Public Health Department for malaria control data. In memoriam Javier Benzaquen Garcia, a young Loreto malariologist (died in October 1998).

Support for this work was provided by a National Foundation for Infectious Diseases-Merck Postdoctoral Fellowship in Emerging Infectious Diseases (Dr. Witzig).

Dr. Javier Aramburú Guarda is chairman of Surveillance of Infectious Diseases, General Office of Epidemiology, Ministry of Health of Perú. His research focuses on antimalarial resistance, integrated malaria control, and malaria control policies.

### References

1. Peru National Malaria Control Program Data. Lima, Peru: Instituto Nacional de Salud; 1997.
2. Malaria Control Program. Malaria statistics 1992-1997. Iquitos, Loreto, Peru: Loreto Department of Public Health; 1998.
3. Gilles HM, Warrell DA. Bruce-Chwatt's essential malariology, 3rd ed. London: Edward Arnold; 1993. p. 129,136.
4. Status of malaria programs in the Americas: XLIV Report. Washington: Pan American Health Organization; 1996 Sep. Document No.: CD39/INF/2.
5. Malaria: epidemiological data, 1990-1992. Geneva: World Health Organization; 1997. Available from: URL: http://www.who.org/whosis/malinfo/8-epidat.htm.
6. Health conditions in the Americas. Vol. II. Geneva: World Health Organization; 1994 Scientific publication No.: 549.
7. Root F. Studies on Brazilian mosquitoes. I. The anophelines of the Nyssorhynchus group. American Journal of Hygiene 1926;6:648-717.
8. Branquinho MS, Lagos CBT, Rocha RM, Natal D, Barata JMS, Cochrane AH, et al. Anophelines in the state of Acre, Brazil, infected with *Plasmodium falciparum, P. vivax*, the variant *P. vivax* VK247 and *P. malariae.* Trans Roy Soc Trop Med Hyg 1993;87:391-4.
9. Need JT, Rogers EJ, Phillips IA, Falcon R, Fernandez R, Carbajal F, et al. Mosquitos (Diptera: Culicidae) capture in the Iquitos area of Peru. J Med Entomol 1993;30:634-8.
10. Fernandez R, Carbajal F, Quintana J, Chauca H, Watts DM. Presencia del *A. (N) darlingi* (Diptera: Culicidae), en alrededores de la ciudad de Iquitos, Loreto-Peru. Sociedad Peruana de Enfermedades Infecciosas y Tropicales 1996;5:10-2.
11. Calderón G, Fernández R, Valle J. Especies de la fauna anofelina, su distribución y algunas consideraciones sobre su abundancia e infectividad en el Perú. Revista Peruana de Epidemiología 1995;8:5-23.
12. Calderón G, Curaca A, Llancari J, Napán M, Sipán F. Distribución geográfica de los vectores de la malaria en el Perú. Revista Peruana de Medicina Tropical 1974;2:88-91.
13. Chauca H, Quintana J. Evaluación *in vivo* de la respuesta de *Plasmodium falciparum* a la cloroquina en foco carretera Yurimaguas-Tarapoto (Región Loreto). Revista Peruana de Epidemiología 1993;6:34-9.

14. Colán E, Quintana J, Ferreli R, San Roman E, Rios R. Malaria por *Plasmodium falciparum* en la Amazonia Peruana. Revista de Farmacología y Terapéutica 1993;3:11-6.
15. Navitsky RC, Witzig RS, Quintana Zurita J, Rios M, Aramburú Guarda JS, Gilman RH, et al. In vivo resistance of *Plasmodium falciparum* to pyrimethamine/sulfadoxine in children of the Amazon region of Peru. Am J Trop Med Hyg 1997;57 Suppl:229.
16. WHO. *In vitro* microtest (Mark II) for the assessment of the response of *Plasmodium falciparum* to chloroquine, mefloquine, quinine, sulfadoxine/pyrimethamine, and amodiaquine. Geneva: World Health Organization; 1990 Document No.: MAP/87.2:1-21.
17. Panduro M, Colán E, Witzig R, Quintana J, Chávez R. Estudio *in vitro* e *in vivo* de la respuesta del *P. falciparum* a chloroquina, sulfadoxina/pirimetamina, quinina, y mefloquina en la zona periurbana y rural de Iquitos. Sociedad Peruana de Enfermedades Infecciosas y Tropicales 1997;6:64.
18. Kublin JG, Witzig RS, Shankar A, Quintana J, Aramburú J, Cortese, JF, et al. Molecular assays for surveillance of antifolate resistant malaria in Peru. Lancet 1998;351:1629-30.
19. Huailu C, Wen Y, Wuanmin K, Chongyi L. Large-scale spraying of bednets to control mosquito vectors and malaria in Sichwan, China. Bull World Health Organization 1995;73:321-8.
20. Freeman T, Bradley M. Temperature is predictive of severe malaria years in Zimbabwe. Trans R Soc Trop Med Hyg 1996;90:232.
